# Assessment of a diverse panel of transmitted/founder HIV-1 infectious molecular clones in a luciferase based CD8 T-cell mediated viral inhibition assay

**DOI:** 10.3389/fimmu.2022.1029029

**Published:** 2022-12-01

**Authors:** Natalia Fernandez, Peter Hayes, Julia Makinde, Jonathan Hare, Deborah King, Rui Xu, Ola Rehawi, Allison T. Mezzell, Laban Kato, Susan Mugaba, Jennifer Serwanga, James Chemweno, Eunice Nduati, Matt A. Price, Faith Osier, Christina Ochsenbauer, Ling Yue, Eric Hunter, Jill Gilmour, The IAVI protocol C investigators

**Affiliations:** ^1^ IAVI Human Immunology Laboratory, Imperial College, London, United Kingdom; ^2^ IAVI, New York, NY, United States; ^3^ Emory Vaccine Center at Yerkes National Primate Research Center, Emory University, Atlanta, GA, United States; ^4^ University of Alabama at Birmingham, Birmingham, AL, United States; ^5^ Uganda Virus Research Institute, Entebbe, Uganda; ^6^ Medical Research Council, Uganda Virus Research Institute and London School of Hygiene and Tropical Medicine Uganda Research Unit, Entebbe, Uganda; ^7^ Kenya Medical Research Institute (KEMRI) Wellcome Trust Research Programme, Kilifi, Kenya; ^8^ Department of Epidemiology and Biostatistics, University of California at San Francisco, San Francisco, CA, United States; ^9^ Department of Infectious Diseases, Imperial College, London, United Kingdom

**Keywords:** HIV, CD8 T-cells, viral inhibition, infection, T-cell response, transmitted founder, infectious molecular clones

## Abstract

**Introduction:**

Immunological protection against human immunodeficiency virus-1 (HIV-1) infection is likely to require both humoral and cell-mediated immune responses, the latter involving cytotoxic CD8 T-cells. Characterisation of CD8 T-cell mediated direct anti-viral activity would provide understanding of potential correlates of immune protection and identification of critical epitopes associated with HIV-1 control.

**Methods:**

The present report describes a functional viral inhibition assay (VIA) to assess CD8 T-cell-mediated inhibition of replication of a large and diverse panel of 45 HIV-1 infectious molecular clones (IMC) engineered with a *Renilla reniformis* luciferase reporter gene (LucR), referred to as IMC-LucR. HIV-1 IMC replication in CD4 T-cells and CD8 T-cell mediated inhibition was characterised in both ART naive subjects living with HIV-1 covering a broad human leukocyte antigen (HLA) distribution and compared with uninfected subjects.

**Results & discussion:**

CD4 and CD8 T-cell lines were established from subjects vaccinated with a candidate HIV-1 vaccine and provided standard positive controls for both assay quality control and facilitating training and technology transfer. The assay was successfully established across 3 clinical research centres in Kenya, Uganda and the United Kingdom and shown to be reproducible. This IMC-LucR VIA enables characterisation of functional CD8 T-cell responses providing a tool for rational T-cell immunogen design of HIV-1 vaccine candidates and evaluation of vaccine-induced T-cell responses in HIV-1 clinical trials.

## Introduction

Immunological protection against infection with human immunodeficiency virus-1 (HIV-1) is likely to require both humoral and cell-mediated immune responses (1–4) with cytotoxic CD8 T-cells being a key component of the cellular immune response to viral infection (4–6). Unlike the antibody field where several functional assays are available to assess the breath and potency of antibodies for vaccine design and assessment (7–9), the CD8 T-cell field has not had such a tool to date. Rather than assessing surrogates of potentially anti-viral T-cell functions, such as cytokine production in response to HIV-1 peptides, characterisation of T-cell mediated direct anti-viral activity provides a more direct understanding of potential correlates of CD8 T-cell efficacy and identification of functional epitopes and proteins targeted by T-cell that are associated with control of viral replication, thereby providing tools for rational T-cell immunogen design, development of effective vaccine candidates and predictive functional assays to assess future HIV vaccine candidates. Previous studies have shown that unlike the interferon (IFN)-γ enzyme-linked immunospot (ELISpot) and flow cytometry assays currently used to assess HIV vaccine candidates (10–12), the VIA correlates with *in vivo* virus control (13–18) and demonstrates the ability of the Merck’s adenovirus type-5 (Ad5) vector to suppress only vaccine matched viruses (19).

Previously we and others developed and applied a functional viral inhibition assay (VIA) to assess CD8 T-cell mediated inhibition of HIV-1 replication in studies of HIV-1 pathogenesis (16, 20) and also in HIV-1 vaccine trials, including phase I and efficacy trials (19, 21–23). In these assays HIV-1 replication and productive infection in autologous CD4 T-cells was assessed by measuring HIV-1 p24 protein release into culture supernatants with inhibition shown to be major histocompatibility complex (MHC) class I dependent. A less labour-intensive modified VIA assessed CD8 T-cell-mediated inhibition of replication of HIV-1 infectious molecular clones (IMC) engineered with a *Renilla reniformis* luciferase reporter gene (LucR), referred to as IMC-LucR VIA (24–29). IMC-LucR luciferase activity in cell cultures measured by light emission was shown to positively correlate with productive HIV-1 replication measured by p24 release (25). This novel IMC-LucR VIA allows for the assessment of CD8 T-cell-mediated inhibition with a larger panel of IMC compared to the previous method.

HIV-1 vaccine candidates designed to elicit HIV-1 specific T-cell responses and tested in STEP/Phambili (30–32), HVTN505 (33), and HVTN 705 (34) efficacy studies were shown to be ineffective in preventing HIV-1 transmission. One possible reason for such failure is the narrow breadth of the vaccine induced T-cell response, with an average of 1-2 epitopes recognised per volunteer. Assessment of inhibition of HIV-1 replication mediated by vaccinees’ CD8 T-cells *in vitro*, demonstrated that the only effective inhibition was observed towards HIV-1 isolates closely matched to the HIV-1 sequences inserted into the vaccine vectors (19). Although these regimens did not protect against HIV-1 transmission and showed limited reduction in viremia in vaccinees who subsequently acquired HIV-1, T-cell responses to 3 or more HIV-1 Gag peptides in STEP vaccinees who acquired HIV-1 were associated with a half-log reduction in viremia, compared with subjects without Gag responses (35, 36). Future vaccine candidates designed to elicit cellular responses would no doubt have a higher chance of success if HIV-1 specific CD8 T-cells were elicited that were able to recognise and inhibit a wider breadth of circulating HIV-1 isolates.

Within the context of studies of the cellular response, the immense sequence diversity of world-wide circulating HIV-1 isolates coupled with the significant virus bottleneck during transmission (37–39) would warrant an assessment of the ability of T-cells to recognise and respond to this global diversity and focus on transmitted/founder (T/F) viruses in studies of HIV-1 pathogenesis and in novel immunogen design for humoral and cellular vaccination. We have addressed this diversity within the VIA by developing a diverse panel of HIV-1 IMC-LucR of different clades and derived from different risk groups and geographic regions to enhance coverage of transmitted and circulating HIV-1 sequences as potential targets for vaccine elicited immune responses. The approach for characterising these panels drew inspiration from the strategy employed in developing HIV pseudovirus panels for identifying and characterising broadly neutralising HIV-1 antibodies (26, 27, 29, 40–44). IMC-LucR representing HIV-1 T/F viruses derived from subjects with early acquisition of HIV-1 (Protocol C) (45–47) were assessed within the IMC-LucR VIA for their ability to infect and replicate within PBMCs derived from both HIV-1 uninfected volunteers and from ART naive subjects living with HIV, selected for their broad coverage of human leukocyte antigen (HLA) haplotypes present with the population and identified as having an ability for virus control (28, 45, 48).

The selection of appropriate and diverse HIV-1 IMC-LucR panels that reflects circulating T/F virus diversity will enable meaningful characterisation of efficacious CD8 T-cell responses, thereby facilitating both rational design of vaccine candidates and their evaluation in clinical trial.

## Materials and methods

### Subjects

Subjects were drawn from IAVI’s Protocol C, a large prospective cohort of 613 volunteers living with HIV (45). Briefly, PBMCs from 13 Protocol C cohort ART naive subjects living with HIV-1 from Uganda, Rwanda, Zambia, and South Africa were selected based on their controller status and Class I HLA allele phenotype to provide bi-specific antibody expanded CD4 and CD8 T-cells to characterise both virus replication and any inhibition of replication, respectively. The characteristics of these 13 volunteers including their plasma viral loads, CD4 T-cell count, estimated time from infection, gender, country, controller status and Class I HLA distribution has been described in detail elsewhere (28, 48, 49).

### Cell lines

293T/17 [HEK 293T/17] cells were obtained from the American Type Culture Collection (ATCC CRL-11268). TZM-bl dual-reporter cell line was obtained through the NIH AIDS Reagent Program, Division of AIDS, NIAID, NIH: TZM-bl ARP-8129, contributed by Dr. John C. Kappes, Dr. Xiaoyun Wu and Tranzyme Inc. Both cell lines were maintained in Dulbecco’s modified Eagle’s medium (DMEM) with 10% V/V HIFCS, 10 mM Hepes, 100 U/mL penicillin and streptomycin (all Sigma, UK) at 37°C ± 5% CO_2_.

### HIV-1 transmitted founder identification and T/F derived infectious molecular clone construction

The construction of T/F IMCs CH077.t (GenBank JN944941), CH106.c (GenBank JN944942) (24), CH505.s (50) and ZM247F_V2 (51) have been described previously. The construction of the recombinant IMC pNL4-3 (52) and IMCs from Protocol C isolates UG.191947 and UG.191882 have also been described previously (53). For plasma viruses from the remaining acutely infected individuals from Protocol C, near full-length single genome amplicons (NFLSGA) were sequenced within approximated 30 days from infection using PacBio^®^ single molecule, long-read sequencing (Pacific Biosciences, Menlo Park, CA, USA) and the Multilayer Directed Phasing and Sequencing (MDPSeq) algorithm (54). Transmitted/founder (T/F) viral sequences were defined in Geneious v9.1.8 (Biomatters, Aukland, NZ) as described previously (46, 47, 55). Two methods of IMC cloning were utilised; for the majority, near full-length amplicons corresponding to the T/F virus sequence and derived using the Q5^®^ High-Fidelity DNA Polymerase (New England Biolabs, MA, USA) were cloned directly at Emory University, Atlanta, USA as described previously (55, 56). The remaining clones were assembled using In-Fusion HD cloning at Emory University, Atlanta, USA and University of Alabama, Birmingham, USA (UAB) from three chemically synthesised fragments (GenScript Biotech, NJ, USA) representing the entire T/F virus sequence with two long terminal repeat (LTRs) as described previously (47). Biological properties (replication capacities, neutralisation sensitivity) of the Rwandan IMCs are described elsewhere (Yue et al., manuscript in preparation).

### Construction of *Renilla* luciferase reporter IMC for VIA

We have previously described the generation of replication-competent *Renilla* luciferase reporter-expressing HIV-1 IMC (24, 25, 27, 29) and the subsequent optimisation utilising a modified encephalomyocarditis virus (EMCV) internal ribosome entry site (IRES) element, collectively referred to as IMC-LucR.6ATRi, including CH077.t-LucR.6ATRi (K4472) and CH505.s-LucR.6ATRi (K4474) (26, 27). To complement the panel of T/F IMC described above, corresponding IMC-LucR.6ATRi were constructed at UAB, using essentially the same molecular design for the insertion of the reporter cassette between the *env* and *nef* open reading frames (ORFs). IMC-LucR.6ATRi proviral plasmids were derived by one of two approaches utilising In-Fusion HD cloning: either by inserting the LucR.6ATRi cassette into an already constructed T/F IMC proviral plasmids derived as described above; or by assembling three chemically synthesised fragments (GenScript Biotech, NJ, USA) representing the entire T/F virus sequence with two LTRs similarly as described previously, but after first inserting the LucR.6ATRi reporter cassette into the 3rd fragment encompassing *env* and *nef* ORFs and 3’ LTR. Resulting IMC-LucR.6ATRi proviral plasmids are named by their IMC ID, followed by the extension “-LucR.6ATRi”, e.g., p.CH106.c-LucR.6ATRi, or p.UG.191996-LucR.6ATRi (**Supplementary Table 1**).

### Generation and characterisation of HIV-1 IMC-LucR

Virus stocks of HIV-1 IMC-LucR clones were generated by proviral DNA transfection of 293T/17 cells as previously described (24, 25, 57). Briefly, 3-5×10^6^ 293T/17 cells were seeded in a T75 culture flask one day prior to transfection. Alternatively, 293T/17 cells were seeded into 6-well plates at 5x10^5^ cells per well. 12μg of DNA in DMEM was transfected using Fu GENE 6 (Promega Ltd., UK), according to manufacturer’s instructions. After 6 hours, transfection medium was replaced with fresh medium. Viral supernatants were harvested and filtered through 0.45μm filters at 48-60 hours post-transfection, and aliquots were frozen at -80°C.

The fifty-percent Tissue-Culture Infectious Dose (TCID50) per mL of the generated virus stocks were determined as previously described (25, 57). Serial dilutions of viruses were added to a 96-well flat bottom plates in quadruplicate followed by 100,000 TZM-bl cells in the presence of 10 μg DEAE-Dextran (Merck Life Science Ltd., UK) per mL. Plates were incubated at 37°C ± 5% CO_2_ for 48 hours and 100 μL culture medium was carefully removed from each well and replaced with luciferase reporter gene assay system reagent (BriteLite Plus Assay System, PerkinElmer Ltd., UK). Luminescence was measured after 3 minutes incubation using a Tecan Infinite M200Pro plate reader (Tecan Ltd., Switzerland).

Alternatively, the virus stocks were also titered on the TZM-bl reporter cell line to determine infectious units (IU)/mL by enumeration of β-galactosidase (β-gal)-stained cells as described previously (24, 58).

### IMC-LucR viral inhibition assay

The CD4 and CD8 T-cell populations were polyclonally expanded from PBMC by culture for 7 days in RPMI 1640 medium with 10% HIFCS and 50 units per ml interleukin-2 (IL-2) (R10/50) and with 0.5 μg/mL CD3/CD8 and CD3/CD4 bi-specific antibody respectively (Professor Johnson Wong, Harvard Medical School, USA). On day 7, 1x10^6^ viable CD4 T-cells were centrifuged in 15mL tubes at 250g for 10 min and supernatants decanted with most of the residual volume aspirated. Cells were infected with HIV-1 IMC-LucR.6ATRi viruses added at a multiplicity of infection (MOI) of 0.1 and spinoculated at 1800g for 2 hours at ambient temperature. Cells resuspended at 1x10^6^ cells/mL in R10/50 and 100 μL CD4 T-cells (100,000 cells) were placed in duplicate wells of a 96-well flat bottom white plate. On the same day, expanded CD8 T-cell were resuspended at 1x10^6^ cells/mL in fresh R10/50 and cultured in 24-well culture plates. Both the CD8 and infected CD4 T-cells were incubated in parallel at 37°C/5% CO_2_ for 3 days. CD8 T-cells were then recovered, centrifuged at 250g/10 min, supernatant decanted and resuspended in an equal volume of R10/50. 100μL CD8 T-cells or R10/50 media were added to the infected CD4 T-cells in duplicate and incubated for an additional 5 days. On day 8 post-CD4 T-cell infection, 100 μL of supernatant were carefully removed from wells and 100 μL Renilla-Glo™ Luciferase Assay System (Promega Ltd., UK) added and incubated in the dark at room temperature for 3 minutes on a rocking platform. Luminescence activity was measured using a Tecan Infinite M200Pro plate reader (Tecan Ltd., Switzerland). Luciferase activity in relative light units (RLU) was determined as a measure of viral replication. CD8 T-cell-mediated inhibition was expressed as log_10_ reduction in RLU of CD4/CD8 T-cell co-cultures, compared with cultures of infected CD4 T-cells alone. A value of ≥0.8 log_10_ inhibition was considered positive (25).

### *In silico* assessment of epitope coverage

To evaluate the contribution of each virus sequence, the proteome files were assessed using an *in-silico* algorithm. The use of CD8 T-cell epitope prediction tools to assess predicted epitope diversity by assigning a coverage gain value to each sequence has been previously described for both HIV-1 (48, 59) and SARS-CoV-2 (60). Whereas previously these values were used to rank each virus proteome for the coverage it provides within the sample population it would also be possible to evaluate specific virus proteomes for the coverage they offer versus a larger population.

Briefly, for each virus proteome a NetMHCpan simulation is performed against distinct Human Leukocyte Antigen (HLA) sequences and the resulting files for each virus proteome are then filtered to extract the peptide, HLA, and rank binding where the rank binding is ≤ 2. These data are then loaded into a PostgreSQL database where an analysis tool is implemented in SQL stored procedures to identify key peptides which appear in at least X viruses’ strains. The conservation metric X is defaulted to 2.2% of the total number of viruses initially being analysed. The analysis tool then selects the virus that contributes the highest number of these key peptides. The selected virus and associated key peptides are then removed from the process and the next virus that contributes the next highest of the remaining key peptides is selected and so on.

### HIV-specific CD4 and CD8 T-cell lines

As with other functional assays, the inclusion of suitable controls for use as a benchmark of assay performance is critical, particularly for application to clinical trials. Larger scale cultures of CD8 T-cell lines enriched for recognition of HIV-1 peptides and autologous CD4 T-cell lines were generated (Makinde et al., manuscript in preparation) from a recipient of a HIV-1 vaccine candidate enrolled into a phase I, randomised, double-blind, placebo-controlled clinical trial (IAVI B001, NCT00851383) conducted to assess the safety and immunogenicity of escalating doses of two recombinant replication defective Adenovirus type-35 (Ad35) vectors containing HIV-1 subtype A gag, reverse transcriptase, integrase and nef genes (Ad35-GRIN) and HIV-1 subtype A env (Ad35-ENV). The trial enrolled 56 healthy HIV-uninfected adults (61).

B001 vaccine recipients were assessed for the breadth and specificity of T-cell responses to peptides matched to the HIV-1 sequence insert used for this vaccine regimen (21). ELISpot peptide matrices were used to identify peptides recognised by each vaccinee. Donor 139 from this B001 study responded to overlapping peptides PPIPVGNIYKRWIIL (HIVgag_64_), VGNIYKRWIILGLNK (HIVgag_65_) and YKRWIILGLNKIVRM (HIVgag_66_) containing the KRWIILGLNK epitope found within the p24 region of HIV-1 Gag protein presented by the HLA B*2705 allele.

Polyclonal HIV-1-specific CD8 T-cell lines and matched CD4 T-cell lines were generated from peripheral blood mononuclear cells of donor 139 as follows. For Gag-specific CD8 T-cells, blood cells were stimulated with HIVgag64-66 peptides at 1 µg/mL prior to surface labelling with IFN-γ and TNF-α reagent conjugated to Phycoerythrin (Miltenyi Biotech) according to the manufacturer’s instructions. Antigen-specific cells were sorted on a FACS Aria II (BD biosciences), and grown in R10 media (RPMI 1640 medium containing 10 mM Hepes 2 mM L-glutamine, 1 mM sodium pyruvate, 100 units/mL penicillin, 0.1 mg/mL streptomycin and 10% v/v heat inactivated fetal bovine serum, all from Sigma, UK) supplemented with 100units/mL recombinant human IL-2 (Peprotech UK), 12.5ug/mL recombinant human IL-15 (Biolegend USA), 1% Human serum (Sigma, UK), 1ug/mL Phytohemagglutinin (PHA) (Sigma, UK), and containing mixed irradiated allogeneic feeders from three unrelated donors. After 5 days in culture cell lines were maintained in the same media with the exclusion of the allogeneic feeder cells, and PHA, and cryopreserved at Day 20. Donor 139 CD4 T-cells were isolated using a Miltenyi Biotech CD4 T-cell isolation kit and expanded in the same way as the CD8 T-cell line. Prior to assay set up, frozen vials of CD4 and CD8 T-cell lines were thawed and washed in 10 ml of R0 (RPMI 1640 medium containing 10 mM Hepes, 2 mM L-glutamine, 1 mM sodium pyruvate, 100 units/mL penicillin and 0.1 mg/mL streptomycin, all from Sigma, UK) supplemented with 10 uL of Benzonase ^®^ (Novagen, Denmark), and rested overnight in R20 (RPMI 1640 medium containing 10 mM Hepes, 2 mM L-glutamine, 1 mM sodium pyruvate, 100 units/mL penicillin and 0.1 mg/mL streptomycin and 20% v/v heat inactivated fetal bovine serum, all from Sigma, UK) prior to use in the assay.

### Statistical analysis

Data were analysed using GraphPad Prism version 9.3.1 for Windows, GraphPad Software, San Diego, California USA.

A two-tailed non-parametric Spearman test was used to compute correlation coefficients between two datasets. A two-tailed non-parametric Wilcoxon matched pairs signed rank test was used to determine significant differences between two sets of paired values. A non-parametric Mann-Whitney test was used to determine significant differences between two sets of unpaired values. Raw data values were unadjusted for all statistical analyses. The threshold for significance was defined as p < 0.05.

### Ethics

Work was approved by the local ethics review boards, including the Rwanda National Ethics Committee, the Uganda Virus Research Institute Science and Ethics Committee (currently the UVRI Research Ethics Committee) and the Uganda National Council of Science and Technology, the University of Cape Town Health Science Research and Ethics Committee, the University of Zambia Research Ethics Committee, and the Emory University Institutional Review Board. Written informed consent was obtained from all participants.

## Results

### Generation of infectious stocks of HIV-1 IMC-LucR and replication in CD4 T-cells

45 infectious molecular clone LucR virus stocks (IMC-LucR) were successfully produced to greater than 10^5^ TCID50 per mL by transfection with IMC-LucR plasmids.

**Figure 1:**
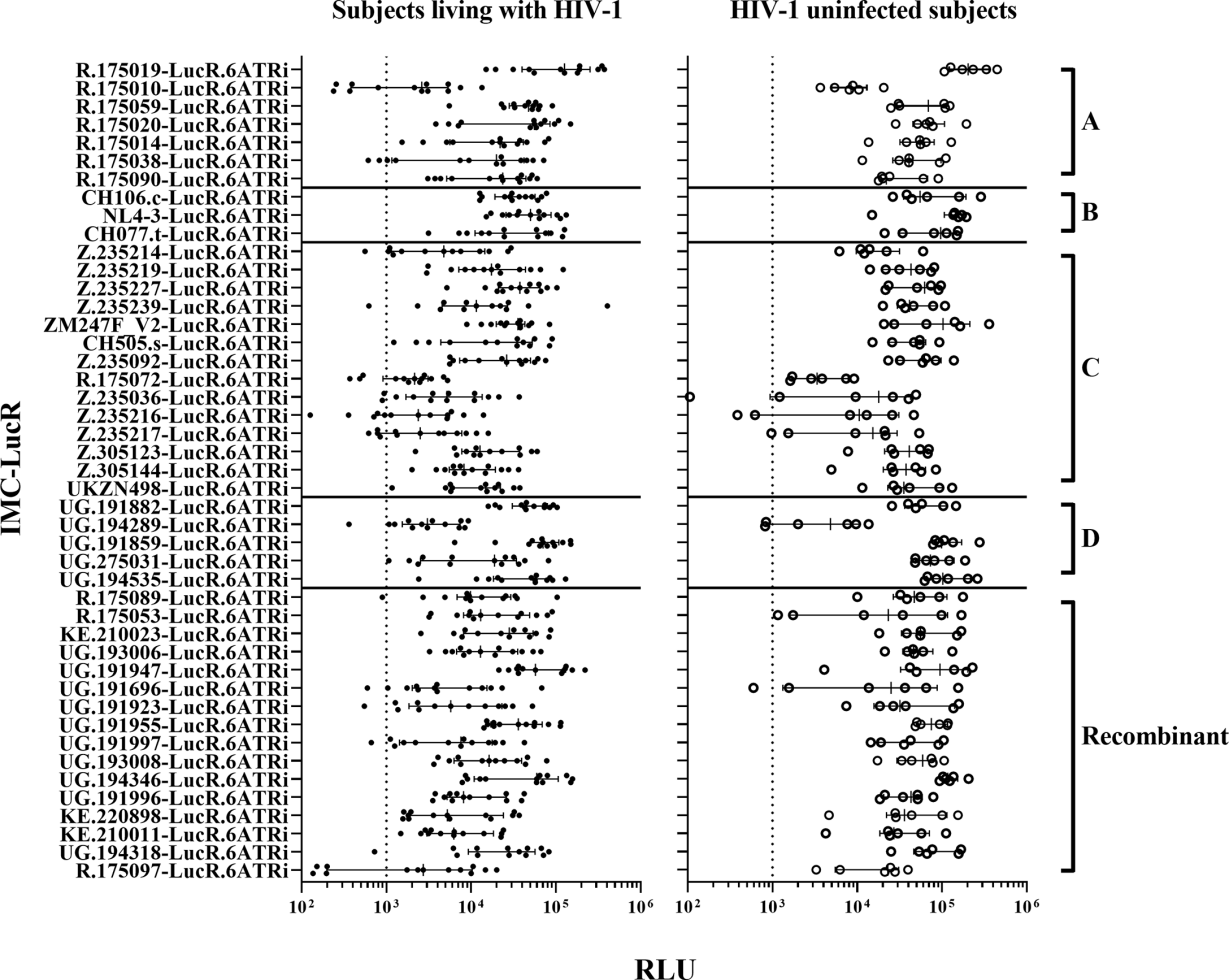
HIV-1 IMC-LucR replication in expanded CD4 T-cells. Replication in expanded CD4 T-cells from ART naive subjects living with HIV-1 (on the left panel), and HIV-1 uninfected subjects (on the right panel) arranged by IMC HIV-1 clade **(A-D)** and Recombinant). Replication is measured as RLU of CD4 T-cell cultures at 8 days post-infection. Each data point represents the mean RLU of duplicated values for each subject’s CD4 T-cells infected with a specific HIV-1 IMC-LucR. Bars represent RLU median with interquartile range. The dotted line represents the assay optimal RLU (>1000).

**Figure 2:**
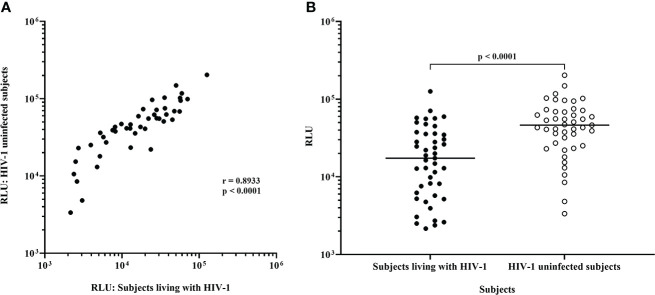
Replication of HIV-1 IMC-LucR in expanded CD4 T-cells from ART naive subjects living with HIV-1 or HIV-1 uninfected subjects. **(A)** Spearman correlation between replication of HIV-1 IMC-LucR in expanded CD4 T-cells from ART naive subjects living with HIV-1 or HIV-1 uninfected subjects. **(B)** Scatter plot of the same data with medians of the groups (bar) and p value generated with a Wilcoxon test. Each data point represents the median RLU of an IMC-LucR replicating in expanded CD4 T-cells from ART naive subjects living with HIV-1 or HIV-1 uninfected subjects.

Following infection at an MOI of 0.1, all 45 IMC-LucR replicated in CD4 T-cells expanded from PBMC from both ART naive subjects living with HIV-1 (**Figure 1**, left panel) and HIV-1 uninfected subjects (**Figure 1**, right panel) determined by luminescence in relative light units (RLU) as a result of LucR protein expression following 8 days of culture of infected CD4 T-cells. Comparing median paired IMC-LucR RLU values for replication in CD4 T-cells from either ART naive subjects living with HIV-1 or uninfected subjects demonstrated a strong positive correlation (r = 0.8933, p<0.0001, Spearman correlation, **Figure 2A**), although RLU values were significantly higher for CD4 T-cells from uninfected subjects (mean 54541, median 46343, IQR 26119 – 70267) compared with from ART naive subjects living with HIV-1 (mean 24750, median 17343, IQR 5988 – 35819) (p <0.0001, Wilcoxon test, **Figure 2B**).

Plasmids for 3 of these 45 IMC-LucR: UG.191859-LucR.6ATRi (clade D), UG.191997-LucR.6ATRi and UG.194318-LucR.6ATRi (Recombinant) could not be produced in sufficient quantities to allow inclusion in future studies on a consistent basis.

Since RLU values of 1000 or more for HIV-1 infected CD4 T-cells is necessary to allow detection of any reduction in RLU values following co-culture with autologous CD8 T-cells, 7 of 45 IMC-LucR were excluded from further study. These viruses [R.175010-LucR.6ATRi, R.175038-LucR.6ATRi (clade A), R.175072-LucR.6ATRi, Z.235036-LucR.6ATRi, Z.235216-LucR.6ATRi, Z.235217-LucR.6ATRi (clade C) and R.175097-LucR.6ATRi (Recombinant)] replicated with RLU values below 1000 in CD4 T-cells from more than 2 ART naive subjects living with HIV-1. Three of these viruses (Z.235036-LucR.6ATRi, Z.235216-LucR.6ATRi and Z.235217-LucR.6ATRi) also exhibited lower RLU values for some HIV uninfected subjects.

Thus, for 35 of these IMC-LucR, stocks were successfully produced with an optimal TCID50 and replicated consistently in CD4 T-cells from both subject groups above a 1000 RLU value. Therefore, 35 of the 45 HIV-1 IMC-LucR were further assessed for studies of CD8 T-cell-mediated inhibition.

### HIV-1 inhibition by CD8 T-cells

**Figure 3:**
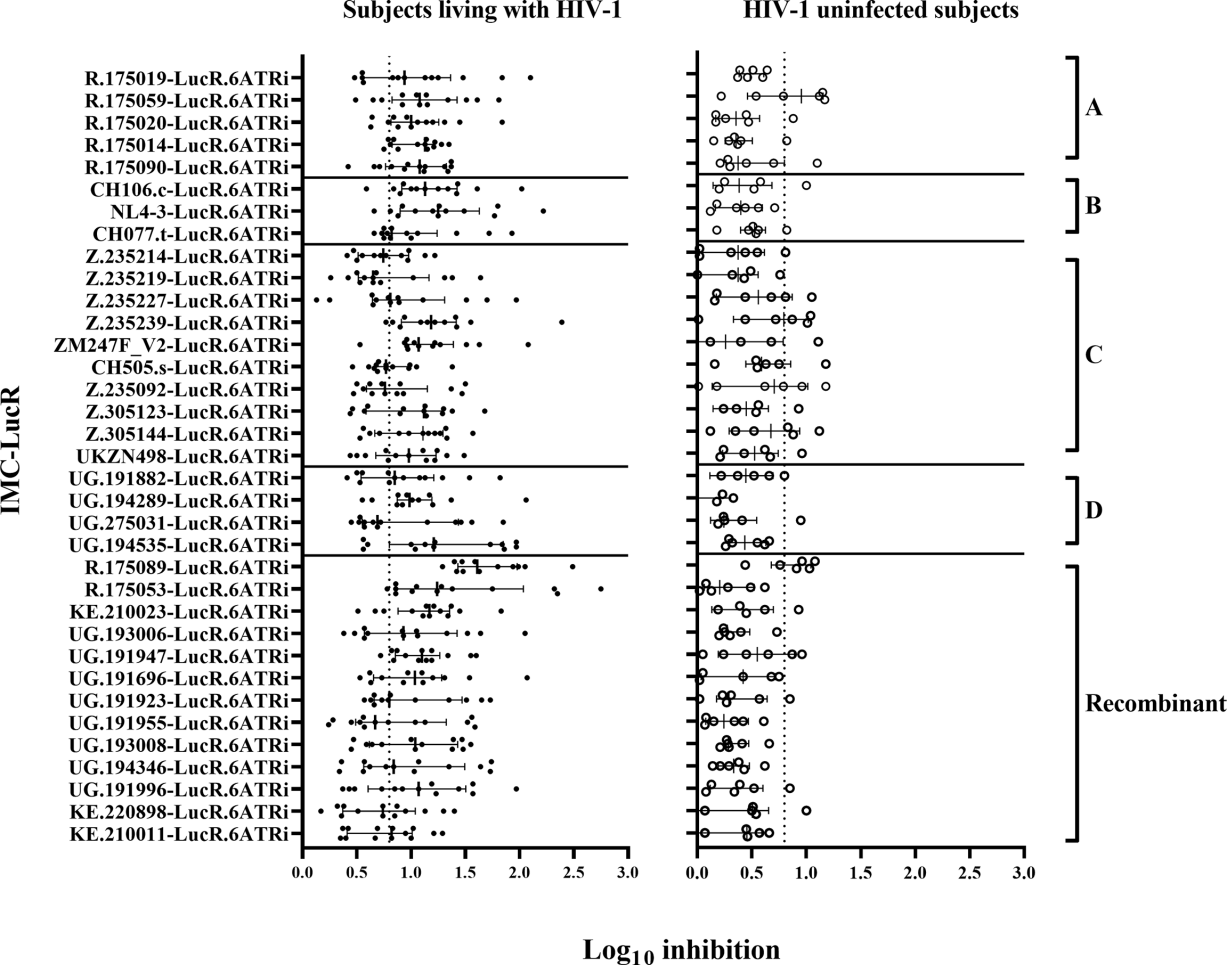
CD8 T-cell-mediated inhibition of HIV-1 replication in autologous CD4 T-cells. Inhibition in T-cell cultures from ART naive subjects living with HIV-1 (on the left panel), and HIV-1 uninfected subjects (on the right panel) arranged by IMC HIV-1 clade **(A-D)**, and Recombinant). Inhibition is measured as log_10_ reduction RLU of CD4 and CD8 T-cell co-cultures compared with infected CD4 T-cells alone. Each data point represents the mean RLU of duplicated values for each CD4 T-cell infected with a specific HIV-1 IMC-LucR. Bars represent RLU median with interquartile range. The dotted line represents the assay positivity value (>0.8 log_10_).

There was a wide range of values for CD8 T-cell-mediated inhibition of replication of the 35 IMC-LucR in autologous CD4 T-cells. All 35 IMC-LucR were inhibited by CD8 T-cells above the 0.8 log_10_ value for at least 4 of the 13 ART naive subjects living with HIV-1 (**Figure 3**, left panel). HIV-1 IMC-LucR R.175089-LucR.6ATRi (Recombinant) was inhibited by CD8 T-cells from all subjects tested. Median log_10_ inhibition values for each IMC-LucR across the 13 ART naive subjects living with HIV-1 ranged from 0.650 to 1.610.

**Figure 4:**
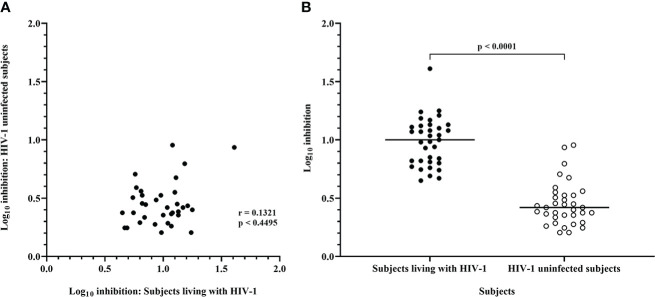
Inhibition mediated by CD8 T-cells from ART naive subjects living with HIV-1 and HIV-1 uninfected subjects. **(A)** Spearman correlation between inhibition of HIV-1 IMC-LucR in ART naive subjects living with HIV-1 or HIV-1 uninfected subjects. **(B)** Scatter plot of the same data with medians of the groups (bar) and p value generated with a Wilcoxon test. Each data point represents the median log_10_ inhibition of an IMC-LucR mediated by CD8 T-cells from ART naive subjects living with HIV-1 or HIV-1 uninfected subjects.

In comparison with the above data, CD8 T-cells from HIV-1 uninfected subjects mediated much lower levels of inhibition of IMC-LucR replication (**Figure 3**, right panel, and **Figure 4B**, p < 0.0001, Wilcoxon test). Median log_10_ inhibition values for each IMC-LucR across the 6 HIV-1 uninfected subjects ranged from 0.205 to 0.955. Median log_10_ inhibition marginally above the 0.8 log_10_ value was observed for 2 of the 35 IMC-LucR: R.175059-LucR.6ATRi (clade A) and R.175089-LucR.6ATRi (Recombinant) at 0.955 and 0.935 respectively.

There was no significant correlation between median inhibition values for CD8 T-cells derived from ART naive subjects living with HIV-1 and uninfected subjects (r = 0.1321, p = 0.4495, Spearman correlation, **Figure 4A**), however, there were notable instances of higher levels of inhibition of a particular IMC-LucR in both subject groups. The high level of inhibition of IMC-LucR R.175089-LucR.6ATRi mediated by CD8 T-cells from ART naive subjects living with HIV-1 stated above (all 13 subjects inhibiting with median inhibition of 1.61 log_10_) was paralleled by inhibition observed in 4 of the 6 HIV-1 uninfected subjects with a high median level of inhibition at 0.935.

There was no significant correlation between the level of IMC-LucR replication (median RLU) and log_10_ inhibition values both for ART naive subjects living with HIV-1 (r = 0.0937, p = 0.5923) or uninfected subjects (r = 0.06629, p = 0.7052). In other words, higher HIV-1 replication did not provide a bigger target for and thereby allow for greater inhibition by CD8 T-cells. Conversely, there was no evidence that higher HIV-1 replication overcame any CD8 T-cell-mediated inhibition of replication as higher HIV-1 replication was not associated with reduced CD8 T-cell-mediated inhibition within the IMC-LucR VIA cultures.

### Protocol C HIV-1 epitope coverage by HIV-1 IMC-LucR

The use of CD8 T-cell epitope prediction tools to assess predicted epitope diversity by assigning a coverage gain value to each sequence has been previously applied for HIV (McGowan et al., 2021). Whereas previously these values were used to rank each virus proteome for the coverage it provides within the sample population, ordering the sequences of the 35 IMCs identified as being functional within the IMC-LucR VIA and applying the previously identified frequency and binding thresholds to calculate the number of unique epitope/HLA interactions, the contribution of each viral sequence can be determined. These values are used to assign a cumulative epitope coverage gain value to each sequence which enables each virus proteome to be ranked for the coverage it provides within the sample population.

**Figure 5:**
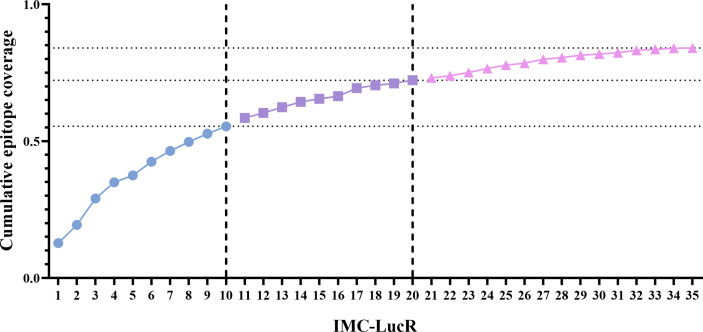
Coverage distribution plot for 35 full length HIV-1 Transmitted/founder proteome sequences, representing the cumulative epitope coverage provided against a larger proteome cohort (N=218). Dotted lines reflect cumulative predictive epitope coverage for each potential panel (identified through dashed lines).

Using this model, the 35 IMC-LucR sequences can be stratified into distinct panels based on the cumulative epitope coverage gain they offer versus the total landscape of predicted CD8 T-cell epitopes within Protocol C (**Figure 5**, **Table 1**). Panel 1 comprises the best 10 IMC-LucR and offers a cumulative epitope coverage of 55.4%; panel 2 comprises the best 20 IMC-LucR and offers a cumulative epitope coverage of 72.3%; whereas panel 3 comprises all 35 IMC-LucR and offers a cumulative epitope coverage of 84.0% against total landscape of predicted CD8 T-cell epitopes within Protocol C. In addition to the cumulative epitope coverage, it is also possible to calculate the individual contribution each IMC is providing to epitope coverage, and this can be used as a confounding metric when evaluating the VIA profile of a particular CD8 T-cell sample using these IMC-LucR panels.

**Table 1:** Cumulative and individual epitope coverage score across the HIV-1 strains.

Number	IMC-LucR Name	Country	Clade	Cumulative Epitope Coverage	Individual Epitope Coverage	GenBank Accession Number*
1	CH077.t-LucR.6ATRi	USA	B	0.1275	0.0311	JN944909
2	UG.191955-LucR.6ATRi	Uganda	A/D	0.1938	0.0302	MW006055
3	ZM247F_V2-LucR.6ATRi	Zambia	C	0.2902	0.0309	FJ496207
4	CH106.c-LucR.6ATRi	USA	B	0.3494	0.0308	JN944897
5	UG.191996-LucR.6ATRi	Uganda	A/D	0.3743	0.0301	MW006056
6	Z.235239-LucR.6ATRi	Zambia	C	0.4245	0.0306	KR820421
7	UG.275031-LucR.6ATRi	Uganda	D	0.4639	0.0305	MW006081
8	Z.305144-LucR.6ATRi	Zambia	C	0.4972	0.0309	N/A
9	NL4-3-LucR.6ATRi	USA	B	0.5273	0.0310	AF324493
10	UG.193008-LucR.6ATRi	Uganda	A/D	0.5538	0.0307	MW006063
11	R.175059-LucR.6ATRi	Rwanda	A	0.5847	0.0222	MT942819
12	UG.191696-LucR.6ATRi	Uganda	A/D	0.6029	0.0212	MW006053
13	R.175090-LucR.6ATRi	Rwanda	A	0.6233	0.0226	MT942927
14	R.175019-LucR.6ATRi	Rwanda	A	0.6431	0.0229	MT942773
15	KE.210011-LucR.6ATRi	Kenya	A/D	0.6548	0.0225	KU749427
16	UG.191947-LucR.6ATRi	Uganda	A/D	0.6640	0.0221	KF716504
17	R.175053-LucR.6ATRi	Rwanda	A/C	0.6936	0.0226	MT942802
18	UG.194535-LucR.6ATRi	Uganda	D	0.7040	0.0224	KF716480
19	Z.235219-LucR.6ATRi	Zambia	C	0.7109	0.0227	KR820366
20	KE.210023-LucR.6ATRi	Kenya	A/C	0.7225	0.0229	KU749429
21	UG.191882-LucR.6ATRi	Uganda	D	0.7307	0.0226	KF716503
22	Z.235214-LucR.6ATRi	Zambia	C	0.7388	0.0228	KR820323
23	R.175020-LucR.6ATRi	Rwanda	A	0.7506	0.0223	MT942776
24	UG.191923-LucR.6ATRi	Uganda	A/D	0.7656	0.0223	MW006054
25	CH505.s-LucR.6ATRi	USA	C	0.7777	0.0098	N/A
26	R.175014-LucR.6ATRi	Rwanda	A	0.7844	0.0218	MT942748
27	UG.194289-LucR.6ATRi	Uganda	D	0.7989	0.0219	MW006068
28	KE.220898-LucR.6ATRi	Kenya	A/D	0.8051	0.0221	KF716468
29	Z.305123-LucR.6ATRi	Zambia	C	0.8134	0.0226	MT195515
30	UG.193006-LucR.6ATRi	Uganda	A/C/D	0.8180	0.0227	MW006062
31	R.175089-LucR.6ATRi	Rwanda	A/C	0.8230	0.0223	MT942914
32	Z.235227-LucR.6ATRi	Zambia	C	0.8319	0.0220	KR820393
33	UG.194346-LucR.6ATRi	Uganda	A/D	0.8353	0.0224	MW006071
34	Z.235092-LucR.6ATRi	Zambia	C	0.8393	0.0227	MT194496
35	UKZN498-LucR.6ATRi	South Africa	C	0.8403	0.0090	KC424096

*GenBank Accession number representing the proviral DNA sequence of each Transmitted/Founder virus. N/A, Not Available.

### HIV-1 specific CD4 and CD8 T-cell lines as IMC-LucR VIA assay controls

Expanded and cryopreserved HIV-specific CD8 T-cell lines and autologous CD4 T-cell lines are currently used as standardised internal assay controls in each batch of the IMC-LucR VIA. They allow for assessment of intra and inter-laboratory variation in assay performance and may be used in laboratory scientist training and subsequent demonstration of proficiency.

**Figure 6:**
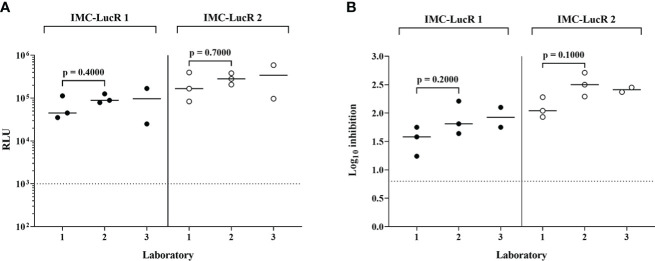
Performance of positive control CD4 and CD8 T-cell lines in the IMC-LucR VIA across 3 laboratories. **(A)** Replication of IMC-LucR in CD4 T-cell line measured by RLU and **(B)** Inhibition of IMC-LucR replication mediated by an autologous CD8 T-cell line measured by log_10_ reduction in RLU of CD4 and CD8 T-cell line co-cultures compared with infected CD4 T-cell line alone. Bars represent median values. Triplicate data for laboratories 1 and 2 were analysed using a Mann-Whitney test. The dotted line represents the assay positivity value (>0.8 log10).

**Figure 6A** displays RLU values for replication of 2 IMC-LucR in the positive control CD4 T-cell line and **Figure 6B** displays log_10_ inhibition mediated by the autologous CD8 T-cell line. Data represents the repeated assays conducted on different occasions at 3 Clinical Research Centres (CRC) situated in Kenya (KEMRI-Wellcome Trust Research Programme), Uganda (MRC/UVRI and LSHTM Uganda Research Unit) and the United Kingdom (IAVI-Human Immunology Laboratory), following initial assay training. Two centres have triplicate assay data and one centre duplicate assay data, the latter therefore, not being included in statistical analyses. There were no significant differences across the two laboratories in either RLU values for replication of IMC-LucR1 and IMC-LucR2 in CD4 T-cells lines (p = 0.4000, p = 0.7000 respectively) or log_10_ inhibition mediated by the CD8 T-cell line (p =0.2000, p = 0.1000 respectively, Mann-Whitney test). RLU and inhibition values obtained by the third centre were similar to values provided by the other two centres.

## Discussion

The immense sequence diversity of HIV-1 continues to be a considerable barrier to the development of an effective vaccine. Understanding the nature of highly potent and broadly cross reactive anti-viral T-cells in both natural HIV-1 infection and following administration of prophylactic vaccine candidates in uninfected subjects, provides a tool for the rational development of T-cell immunogens and assessment of vaccines in clinical studies and studies of somewhat unique subjects, such as those living with HIV-1 who are able to naturally control HIV-1 *in vivo* in the absence of anti-retroviral therapy. An additional consideration is the genetic bottleneck that exists during transmission such that infection is founded in the donor by only 1 or 2 quasi-species from the recipient (39)

The present report is focused on one key aspect of anti-viral immunity, that of the functional anti-viral CD8 T-cell response to HIV-1. The immense diversity of HIV-1 also complicates assessments of such immune responses *in vitro*. Unlike the interferon (IFN)-γ enzyme-linked immunospot (ELISpot) and flow cytometry assays currently used to assess HIV vaccine candidates (10–12), the VIA correlates with *in vivo* virus control (13–18). *In vitro* assessments allowing full identification of HIV-1 epitopes recognised by T-cells requires laboratory assays employing stimuli consisting of very large sets of HIV-1 peptides to capture the full proteome diversity of HIV-1. Our own group has addressed this recently by use of IFN-γ ELISpot assay and a set of 1408 potential T-cell epitope (PTE) peptides to assess HIV-1 epitope recognition by CD8 T-cells derived from ART naive subjects living with HIV-1 (28, 62). Using a smaller but still clade-diverse panel of 10 IMC-LucR, the breadth of CD8 T-cell mediated HIV-1 inhibition in terms of the number of IMC inhibited, was significantly positively correlated with the number of PTE peptides recognised by CD8 T-cells (28).

Complimentary to the ability to determine HIV-1 epitope recognition by CD8 T-cells, is the ability to also assess the extent that these CD8 T-cells may mediate direct inhibition of HIV-1 replication *in vitro*, in this case by use of IMC-LucR in the VIA. Unlike the HIV antibody field where functional antibody neutralisation assays have been critical to identify antibodies with breadth and potency for immunogen design or passive administration (9), the cellular field has not had a functional assay to identify T-cells with breadth and potency against diverse strains of replication competent viruses. Use of diverse HIV-1 IMC-LucR in the VIA paired with identification of the HIV-1 PTE peptides recognised by CD8 T-cells will allow associations to be made between targeting of particular epitopes and the extent of inhibition of HIV-1 IMC containing those epitopes. Such assessments can be made in the context of natural control or lack of control of HIV-1 replication *in vivo* in ART naive subjects living with HIV-1 as well as in the context of studies of therapeutic treatments or structured treatment interruptions designed to potentially elicit or enhance CD8 T-cell responses to HIV-1 in ART naive subjects living with HIV-1 receiving anti-retroviral therapy.

We have addressed diversity of transmitted HIV-1 within the IMC-LucR VIA by selecting a panel of 35 HIV-1 IMC-LucR of different clades and derived from different risk groups and geographic regions to enhance coverage of HIV-1 sequences. In most subjects, acquisition of HIV-1 appears to be due to transmission of a single virus or transmitted/founder (T/F) virus (37, 38). The majority of IMC were TFV isolates derived from subjects with recent HIV-1 acquisition (45–47, 63) with such isolates being those that vaccine elicited immune responses would potentially need to target in order to prevent HIV-1 transmission or limit subsequent HIV-1 replication. The ability of CD8 T-cells from ART naive subjects living with HIV-1 to inhibit these IMC was assessed, with subjects selected with broad coverage of HLA types present with the large protocol C cohort. In this IMC-LucR VIA applied to ART naive subjects living with HIV-1, inhibition of the test IMC-LucR can be clearly determined as use of IMC-LucR overcomes the issue of co-detection of replication of both the input IMC tested and any endogenous HIV-1.

In terms of replication of IMC-LucR in CD4 T-cells, there was a wide range in median RLU values across the initial 45 IMC tested. These RLU values were consistent between CD4 T-cells from ART naive subjects living with HIV-1 and HIV-1 uninfected subjects with a strong and significant positive correlation, indicating levels of replication are primarily due to virus isolate specific factors. However, there was a higher level of HIV-1 replication in CD4 T-cells from uninfected subjects. The potential reasons for this difference in level of HIV-1 replication were not investigated in this study, the most plausible reason being some impairment of CD4 T-cell function in ART naive subjects living with HIV-1 compared with uninfected subjects.

Of the 45 IMC-LucR tested, stocks of 35 IMC-LucR were produced in sufficient amounts and replicated in CD4 T-cells to a high enough level to allow consistent use in IMC-LucR VIA. As expected, CD8 T-cells from ART naive subjects living with HIV-1 mediated efficient inhibition of HIV-1 replication, with all 35 IMC-LucR being inhibited over the 0.8 log_10_ value by at least 4 subjects. CD8 T-cells from HIV-1 uninfected subjects mediated comparatively minimal inhibition of HIV-1 replication, though for two of the 35 IMC-LucR tested median inhibition was over the assay positivity 0.8 log_10_ value. The primary application of IMC-LucR VIA in subjects without HIV-1 infection would be in clinical trials of prophylactic HIV-1 vaccine candidates where such trials include pre-vaccination sampling. The standard approach in the clinical trial setting would be to expand CD4 T-cells only from pre-vaccination PBMC to be used as targets for HIV-1 IMC-LucR and to expand CD8 T-cells from both pre- and post-vaccination time points, allowing clear delineation of any vaccine induced effect.

We have previously demonstrated that the efficient CD8 T-cell-mediated inhibition observed in ART naive subjects living with HIV-1 or uninfected recipients of HIV-1 vaccine candidates requires proximity between CD8 T-cells and infected CD4 T-cells and is reversed by MHC-I blockade (16). However, MHC-I blockade had no effect on any inhibition detected in cultures from HIV-1 uninfected subjects, leading to the conclusion that efficient CD8 T-cell mediated inhibition is due to CD8 T-cell recognition of peptide/MHC-I complexes presented on the surface of infected CD4 T-cells. Any comparatively lower inhibition detected with CD8 T-cells from uninfected subjects would be as a result of a non-specific process in the absence of peptide/MHC-I recognition. Separation of CD4 and CD8 T-cells across trans-well cultures still allowed a low level of CD8 T-cell mediated inhibition indicating that release of soluble factors from CD8 T-cells plays some role (64).

Some limitations remain in the present study and application of this approach.

The use of IMC-LucR simplifies assessment of the level of HIV-1 replication and inhibition and reduces cost compared with use of HIV-1 p24 ELISA. However, the IMC-LucR VIA is still a relatively labour-intensive assay including a 7-day polyclonal expansion of both CD4 and CD8 T-cells prior to CD4 T-cell infection and then culture for a further 8 days under additional laboratory biosafety constraints for work with infectious HIV-1.

Due to the immense diversity of HIV-1, culture and polyclonal expansion of T-cells may be the only means to allow full and detailed assessments of multiple immune parameters with what is typically limited subject blood sampling volumes (28). However, polyclonal T-cell expansion may result in populations of T-cells with phenotypes and functions that are different to those of ex vivo T-cells in PBMC.

We have previously shown that CD3/CD8 bi-specific antibody expansion and traditional phytohemagglutinin/IL-2 stimulation result in similar levels of productive HIV-1 replication in CD4 T-cell cultures measured by p24 protein release (16). CD3/8 and CD3/4 bi-specific antibody expanded CD3+ T-cells consist of 97% CD4 T-cells and 88% CD8 T-cells respectively (16) with CD8 T-cells consisting of both central memory and effector memory phenotypes (19).

We have also previously demonstrated similar function of HIV-1-specific CD8 T-cells in PBMC and expanded CD8 T-cells in IFN-γ production detected by ELISpot and flow cytometric assays (28, 62) and HIV-1 inhibition in VIA (19). Despite demonstrating that expanded CD8 T-cells have similar functionality to unexpanded CD8 T-cells and the IMC-LucR VIA does result in degree of log_10_ inhibition, the ultimate interpretation of VIA data is more suited to a qualitative outcome. In other words, the IMC-LucR VIA assay would answer the question: “does the study subject possess circulating CD8 T-cells that can inhibit HIV-1 replication and if so, what is the breadth of that inhibition in terms of inhibition of different IMC-LucR?”. Such information is then particularly informative if accompanied by identification of HIV-1 epitopes recognised by these CD8 T-cells (28) in the context of subjects with differing rates of HIV-1 disease progression and *in vivo* plasma viral loads. Evaluation of positive log_10_ inhibition is calculated using a standardised, predetermined threshold of 0.8 (25). This value was calculated based on statistical analysis of 2 IMC-LucR’s assessed against PBMC samples for 8 ART naive subjects living with HIV. As the data sets for each IMC-LucR expands, recalculation of this threshold and application of virus specific cut-offs may be prudent.

The present study is not designed to describe in detail the immune response of the 13 ART naive subjects living with HIV-1 but to characterise their CD8 T-cell mediated inhibition of the IMC-LucR tested. Similar approaches by our lab and others demonstrates the potency induced T-cell responses (21, 23). Studies utilising the IMC-LucR VIA, and other assays are underway in our laboratory with a larger number of carefully selected subjects from the protocol C cohort (65) to assess CD8 T-cell responses in the context of differing natural control of viral replication *in vivo*.

Finally, due to the immense diversity of HIV-1, even with 35 IMC-LucR the coverage of all potential predicted HIV-1 epitopes within the protocol C cohort was not complete. However, it does provide a high level of epitope coverage at 84.0%.

HIV-specific CD8 and matched CD4 T-cell lines are currently used for the standardisation of the IMC-LucR VIA facilitating the establishment of the assay across laboratories collaborating centres in Kenya (KEMRI-Wellcome Trust Research Programme), Uganda (MRC/UVRI and LSHTM Uganda Research Unit) and the United Kingdom (IAVI-Human Immunology Laboratory) under the IAVI Clinical Research Centre (CRC) network, providing tools for the successfully laboratory scientist training and technology transfer of this assay, the ultimate demonstration of technical competence and the continued assay stability over time.

HIV-1 replication RLU values and CD8 T -cell inhibition values were comparable both across the 3 laboratories and between assays at each laboratory. Such controls will allow application of the IMC-LucR VIA assay in clinical trials across different centres and comparison of data with confidence. Inclusion of such controls in each batch of samples tested in IMC-LucR VIA, where certain levels of IMC-LucR replication and inhibition are expected, will demonstrate both the reliability of each assay conducted and the derived clinical trial data. Our data demonstrate that despite the IMC-LucR VIA being a complex functional assay, with appropriate training, standard operating procedure (SOP) and reagents, and quality controls it is possible to standardise this assay as a qualitative assay across multiple laboratories in different continents.

In summary, this report describes the generation and viral inhibition potential of a diverse panel of HIV-1 luciferase-labelled infectious molecular clones (IMC-LucR) that have broad representation of previously circulating HIV-1 sequences. Application of these panels would allow assessment of the direct anti-viral function of CD8 T-cells in the viral inhibition assay. IMC-LucR VIA along with other companion assays will allow determination of the breadth of HIV-1 inhibition and identification of effective epitopes targeted by broadly potent anti-viral CD8 T-cells that result in this inhibition. In a similar fashion to the use of the HIV neutralisation assay for the identification of broadly potent and functional antibodies and targets, such assessments of broadly potent and effective T-cells in a functional assay may be applied to study subjects of differing HIV-1 progression rates to inform on both effective targets for virus control and elimination, thereby informing on rational CD8 T-cell vaccine candidate design, as well as more effective evaluation of CD8 T-cell efficacy in both prophylactic and therapeutic clinical trials.

The IMC-LucR VIA is currently being used in the assessment of functional T-cell responses in vaccine recipients in a phase 1 trial of ChAdOx1- and MVA-vectored Conserved Mosaic HIV-1 Vaccines in Healthy, Adult HIV-1-negative Volunteers in Eastern and Southern Africa (HIV-CORE 006, NCT04553016).

In addition, this IMC-LucR VIA will be used to assess antiviral activity of vaccine-induced T-cell responses in several clinical trials: a phase 1/2a open label trial to assess safety and immunogenicity of candidate T-cell vaccines ChAdOx1.HTI and MVA.HTI given sequentially to healthy HIV-1/2 negative adult volunteers in Oxford, UK (HIV-CORE 0051, NCT04563377); a phase 1 dose escalation open label trial to assess safety and immunogenicity of candidate ChAdOx1- and MVA-vectored conserved mosaic HIV-1 vaccines, given sequentially to healthy HIV-1/2-negative adult volunteers in Oxford, UK (HIV-CORE 0052, NCT04586673); and used as an exploratory immunological assay in a subset of participants in a phase 2 randomised, placebo-controlled trial of vedolizumab with or without therapeutic HIV MVA vaccine in individuals who started antiretrovirals during primary or chronic infection (EHVA T02/ANRS VRI07, NCT04120415).

## Data availability statement

The datasets presented in this study can be found in online repositories. The names of the repository/repositories and accession number(s) can be found below: https://dataspace.iavi.org.

## Ethics statement

Work was approved by the local ethics review boards, including the Rwanda National Ethics Committee, the Uganda Virus Research Institute Science and Ethics Committee (currently the UVRI Research Ethics Committee) and the Uganda National Council of Science and Technology, the University of Cape Town Health Science Research and Ethics Committee, the University of Zambia Research Ethics Committee, and the Emory University Institutional Review Board. Written informed consent was obtained from all participants. The patients/participants provided their written informed consent to participate in this study.

## Author contributions

NF, PH were responsible for methodology, data generation and curation, generation of some IMC-LucR, formal analysis, original draft preparation, review, and editing. JM was responsible for methodology, data curation, formal analysis, original draft preparation, review, and editing. JH was responsible for conceptualisation, sample application, data curation, formal analysis, original draft preparation and review. DK was involved in conceptualisation, sample application and review. CO, LY and EH conceptualised the molecular cloning strategy and oversaw the generation, performed by RX, OR and AM, of all the IMC and the majority of IMC-LucR tested in this study. EH was involve in conceptualisation, methodology and original draft review and editing. LK, SM, JS, EN, and JC generated part of the viral inhibition data for this manuscript and were involved in original draft review. MP was one of the lead investigators on IAVI protocol C study and was involved in original draft review. FO contributed to study design and original draft review. JG was one of the lead investigators on IAVI protocol C study, and in the current study was the principal investigator involved in conceptualisation, methodology, data review and original draft review and editing. The IAVI protocol C investigators were responsible for the initiation and successful completion of the Protocol C study. All authors contributed to manuscript revision, read, and approved the submitted version.

## Funding

This work was made possible by IAVI, which is supported by funding from many donors, including the Bill and Melinda Gates Foundation, the Ministry of Foreign Affairs of Denmark, Irish Aid, the Ministry of Finance of Japan in partnership with The World Bank, the Ministry of Foreign Affairs of the Netherlands, the Norwegian Agency for Development Cooperation, the United Kingdom Department for International Development, and the US Agency for International Development. The full list of IAVI donors is available at http://www.iavi.org. Partial funding was also provided by the National Institute of Allergy and Infectious Diseases (NIAID R01 AI51231), National Institutes of Health. The contents of this manuscript are the responsibility of the authors and do not necessarily reflect the views of USAID or the US Government. This project has also received funding from the European Union’s Horizon 2020 research and innovation programme under grant agreement No 681137 and No 681032.

## Acknowledgments

We thank the IAVI protocol C investigators who are: Eduard J. Sanders, Centre for Geographic Medicine-Coast/KEMRI, Kenya and University of Oxford, Oxford, United Kingdom. Omu Anzala, KAVI-Institute of Clinical Research, Nairobi, Kenya. Anatoli Kamali, IAVI, Nairobi, Kenya. Etienne Karita, Center for Family Health Research, Kigali, Rwanda. William Kilembe, Mubiana Inambao and Shabir Lakhi, Center for Family Health Research, Lusaka and Ndola, Zambia. Susan Allen and Eric Hunter, Emory University, Georgia, United States of America. Vinodh Edward, The Aurum Institute, Johannesburg and Rustenburg, South Africa. Pat Fast, IAVI, New York, United States of America. Matt A. Price, IAVI, New York and Department of Epidemiology and Biostatistics, University of California San Francisco, United States of America. Jill Gilmour, IAVI Human Immunology Laboratory, Imperial College, London, United Kingdom. Jianming Tang, School of Medicine, University of Alabama, United States of America. Fran Priddy, IAVI, New York, United States of America. Mary H. Latka, The Aurum Institute, South Africa. Linda-Gail Bekker, Desmond Tutu Health Foundation, University of Cape Town, Cape Town, South Africa. Pontiano Kaleebu, Medical Research Council, Uganda Virus Research Institute and London School of Hygiene and Tropical Medicine Uganda Research Unit (MULS), Entebbe and Masaka, Uganda. The investigators can be contacted *via* Matt A. Price (email MPrice@iavi.org).

## Conflict of interest

The authors declare that the research was conducted in the absence of any commercial or financial relationships that could be construed as a potential conflict of interest.

## Publisher’s note

All claims expressed in this article are solely those of the authors and do not necessarily represent those of their affiliated organizations, or those of the publisher, the editors and the reviewers. Any product that may be evaluated in this article, or claim that may be made by its manufacturer, is not guaranteed or endorsed by the publisher.

## References

[1] McElrath MJ Haynes BF. Induction of immunity to human immunodeficiency virus type-1 by vaccination. Immun (2010) 33(4):542–54. doi: 10.1016/j.immuni.2010.09.011 PMC3031162 21029964

[2] Burton DR Ahmed R Barouch DH Butera ST Crotty S Godzik A. A blueprint for HIV vaccine discovery. Cell Host Microbe (2012) 12(4):396–407. doi: 10.1016/j.chom.2012.09.008 23084910 PMC3513329

[3] Arunachalam PS Charles TP Joag V Bollimpelli VS Scott MKD Wimmers F. T Cell-inducing vaccine durably prevents mucosal SHIV infection even with lower neutralizing antibody titers. Nat Med (2020) 26:932–40. doi: 10.1038/s41591-020-0858-8 PMC7303014 32393800

[4] Collins DR Gaiha GD Walker BD. CD8(+) T cells in HIV control, cure and prevention. Nat Rev Immunol (2020) 20(8):471–82. doi: 10.1038/s41577-020-0274-9 PMC7222980 32051540

[5] Betts MR Nason MC West SM De Rosa SC Migueles SA Abraham J. HIV Nonprogressors preferentially maintain highly functional HIV-specific CD8+ T cells. Blood (2006) 107(12):4781–9. doi: 10.1182/blood-2005-12-4818 PMC1895811 16467198

[6] Goonetilleke N Liu MK Salazar-Gonzalez JF Ferrari G Giorgi E Ganusov VV. The first T cell response to transmitted/founder virus contributes to the control of acute viremia in HIV-1 infection. J Exp Med (2009) 206(6):1253–72. doi: 10.1084/jem.20090365 PMC2715063 19487423

[7] Mascola JR D'Souza P Gilbert P Hahn BH Haigwood NL Morris L. Recommendations for the design and use of standard virus panels to assess neutralizing antibody responses elicited by candidate human immunodeficiency virus type 1 vaccines. J virol (2005) 79(16):10103–7. doi: 10.1128/JVI.79.16.10103-10107.2005 PMC1182642 16051803

[8] Sarzotti-Kelsoe M Bailer RT Turk E Lin CL Bilska M Greene KM. Optimization and validation of the TZM-bl assay for standardized assessments of neutralizing antibodies against HIV-1. J Immunol Methods (2014) 409:131–46. doi: 10.1016/j.jim.2013.11.022 PMC4040342 24291345

[9] Montefiori DC Roederer M Morris L Seaman MS. Neutralization tiers of HIV-1. Curr Opin HIV AIDS (2018) 13(2):128–36. doi: 10.1097/COH.0000000000000442 PMC5802254 29266013

[10] D'Souza MP Altfeld M. Measuring HIV-1-specific T cell immunity: how valid are current assays? J Infect Dis (2008) 197(3):337–9. doi: 10.1086/525288 18184092

[11] Bennett MS Ng HL Ali A Yang OO. Cross-clade detection of HIV-1-specific cytotoxic T lymphocytes does not reflect cross-clade antiviral activity. J Infect diseases (2008) 197(3):390–7. doi: 10.1086/525281 18184090

[12] Valentine LE Piaskowski SM Rakasz EG Henry NL Wilson NA Watkins DI. Recognition of escape variants in ELISPOT does not always predict CD8+ T-cell recognition of simian immunodeficiency virus-infected cells expressing the same variant sequences. J Virol (2008) 82(1):575–81. doi: 10.1128/JVI.00275-07 PMC2224389 17959674

[13] Yang OO Walker BD. CD8+ cells in human immunodeficiency virus type I pathogenesis: cytolytic and noncytolytic inhibition of viral replication. Adv Immunol (1997) 66:273–311. doi: 10.1016/S0065-2776(08)60600-8 9328644

[14] Sáez-Cirión A Lacabaratz C Lambotte O Versmisse P Urrutia A Boufassa F. HIV Controllers exhibit potent CD8 T cell capacity to suppress HIV infection ex vivo and peculiar cytotoxic T lymphocyte activation phenotype. Proc Natl Acad Sci USA (2007) 104(16):6776–81. doi: 10.1073/pnas.0611244104 PMC1851664 17428922

[15] Deeks SG Walker BD. Human immunodeficiency virus controllers: mechanisms of durable virus control in the absence of antiretroviral therapy. Immunity (2007) 27(3):406–16. doi: 10.1016/j.immuni.2007.08.010 17892849

[16] Spentzou A Bergin P Gill D Cheeseman H Ashraf A Kaltsidis H. Viral inhibition assay: a CD8 T cell neutralization assay for use in clinical trials of HIV-1 vaccine candidates. J Infect diseases (2010) 201(5):720–9. doi: 10.1086/650492 20132004

[17] Yang H Wu H Hancock G Clutton G Sande N Xu X. Antiviral inhibitory capacity of CD8+ T cells predicts the rate of CD4+ T-cell decline in HIV-1 infection. J Infect Dis (2012) 206(4):552–61. doi: 10.1093/infdis/jis379 PMC4192045 22711904

[18] Slichter CK Friedrich DP Smith RJ Walsh PN Mize G Czartoski JL. Measuring inhibition of HIV replication by ex vivo CD8^+^ T cells. J Immunol Methods (2014) 404:71–80. doi: 10.1016/j.jim.2013.12.006 24374374 PMC3955096

[19] Hayes PJ Cox JH Coleman AR Fernandez N Bergin PJ Kopycinski JT. Adenovirus-based HIV-1 vaccine candidates tested in efficacy trials elicit CD8+ T cells with limited breadth of HIV-1 inhibition. Aids (2016) 30(11):1703–12. doi: 10.1097/QAD.0000000000001122 27088318

[20] Fauce SR Yang OO Effros RB. Autologous CD4/CD8 co-culture assay: a physiologically-relevant composite measure of CD8+ T lymphocyte function in HIV-infected persons. J Immunol Methods (2007) 327(1-2):75–81. doi: 10.1016/j.jim.2007.07.017 17716683 PMC2151928

[21] Kopycinski J Hayes P Ashraf A Cheeseman H Lala F Czyzewska-Khan J. Broad HIV epitope specificity and viral inhibition induced by multigenic HIV-1 adenovirus subtype 35 vector vaccine in healthy uninfected adults. PloS One (2014) 9(3):e90378. doi: 10.1371/journal.pone.0090378 24609066 PMC3946500

[22] Mutua G Farah B Langat R Indangasi J Ogola S Onsembe B. Broad HIV-1 inhibition *in vitro* by vaccine-elicited CD8(+) T cells in African adults. Mol Ther Methods Clin Dev (2016) 3:16061. doi: 10.1038/mtm.2016.61 27617268 PMC5006719

[23] Ahmed T Borthwick NJ Gilmour J Hayes P Dorrell L Hanke T. Control of HIV-1 replication *in vitro* by vaccine-induced human CD8(+) T cells through conserved subdominant pol epitopes. Vaccine (2016) 34(9):1215–24. doi: 10.1016/j.vaccine.2015.12.021 PMC4769096 26784683

[24] Ochsenbauer C Edmonds TG Ding H Keele BF Decker J Salazar MG. Generation of transmitted/founder HIV-1 infectious molecular clones and characterization of their replication capacity in CD4 T lymphocytes and monocyte-derived macrophages. J Virol (2012) 86(5):2715–28. doi: 10.1128/JVI.06157-11 PMC3302286 22190722

[25] Naarding MA Fernandez N Kappes JC Hayes P Ahmed T Icyuz M. Development of a luciferase based viral inhibition assay to evaluate vaccine induced CD8 T-cell responses. J Immunol Methods (2014) 409:161–73. doi: 10.1016/j.jim.2013.11.021 PMC4236027 24291126

[26] Alberti MO Jones JJ Miglietta R Ding H Bakshi RK Edmonds TG. Optimized replicating renilla luciferase reporter HIV-1 utilizing novel internal ribosome entry site elements for native nef expression and function. AIDS Res Hum Retroviruses (2015) 31(12):1278–96. doi: 10.1089/aid.2015.0074 PMC4663642 26101895

[27] Prevost J Richard J Medjahed H Alexander A Jones J Kappes JC. Incomplete downregulation of CD4 expression affects HIV-1 env conformation and antibody-dependent cellular cytotoxicity responses. J Virol (2018) 92(13):e00484–18. doi: 10.1128/JVI.00484-18 29669829 PMC6002730

[28] Hayes P Fernandez N Ochsenbauer C Dalel J Hare J King D. Breadth of CD8 T-cell mediated inhibition of replication of diverse HIV-1 transmitted-founder isolates correlates with the breadth of recognition within a comprehensive HIV-1 gag, nef, env and pol potential T-cell epitope (PTE) peptide set. PloS One (2021) 16(11):e0260118. doi: 10.1371/journal.pone.0260118 34788349 PMC8598018

[29] Edmonds TG Ding H Yuan X Wei Q Smith KS Conway JA. Replication competent molecular clones of HIV-1 expressing renilla luciferase facilitate the analysis of antibody inhibition in PBMC. Virology (2010) 408(1):1–13. doi: 10.1016/j.virol.2010.08.028 20863545 PMC2993081

[30] Buchbinder SP Mehrotra DV Duerr A Fitzgerald DW Mogg R Li D. Efficacy assessment of a cell-mediated immunity HIV-1 vaccine (the step study): a double-blind, randomised, placebo-controlled, test-of-concept trial. Lancet (2008) 372(9653):1881–93. doi: 10.1016/S0140-6736(08)61591-3 PMC2721012 19012954

[31] McElrath MJ De Rosa SC Moodie Z Dubey S Kierstead L Janes H. HIV-1 vaccine-induced immunity in the test-of-concept step study: a case-cohort analysis. Lancet (2008) 372(9653):1894–905. doi: 10.1016/S0140-6736(08)61592-5 PMC2774110 19012957

[32] Gray GE Allen M Moodie Z Churchyard G Bekker LG Nchabeleng M. Safety and efficacy of the HVTN 503/Phambili study of a clade-b-based HIV-1 vaccine in south Africa: a double-blind, randomised, placebo-controlled test-of-concept phase 2b study. Lancet Infect Dis (2011) 11(7):507–15. doi: 10.1016/S1473-3099(11)70098-6 PMC3417349 21570355

[33] Hammer SM Sobieszczyk ME Janes H Karuna ST Mulligan MJ Grove D. Efficacy trial of a DNA/rAd5 HIV-1 preventive vaccine. N Engl J Med (2013) 369(22):2083–92. doi: 10.1056/NEJMoa1310566 PMC4030634 24099601

[34] Gray GE Mngadi K Lavreys L Luedtke A Nijs S Stieh D. (2022). Phase IIB efficacy trial of mosaic HIV-1 vaccine regimen in African women: Imbokodo [CROI Abstract 121]. Abstracts From CROI 2022 Conference on Retroviruses and Opportunistic Infections. CROI 2022 Abstract eBook. p47.

[35] Janes H Frahm N DeCamp A Rolland M Gabriel E Wolfson J. MRKAd5 HIV-1 Gag/Pol/Nef vaccine-induced T-cell responses inadequately predict distance of breakthrough HIV-1 sequences to the vaccine or viral load. PloS One (2012) 7(8):e43396. doi: 10.1371/journal.pone.0043396 22952672 PMC3428369

[36] Janes H Friedrich DP Krambrink A Smith RJ Kallas EG Horton H. Vaccine-induced gag-specific T cells are associated with reduced viremia after HIV-1 infection. J Infect diseases (2013) 208(8):1231–9. doi: 10.1093/infdis/jit322 PMC3778967 23878319

[37] Keele BF Giorgi EE Salazar-Gonzalez JF Decker JM Pham KT Salazar MG. Identification and characterization of transmitted and early founder virus envelopes in primary HIV-1 infection. Proc Natl Acad Sci U S A (2008) 105(21):7552–7. doi: 10.1073/pnas.0802203105 PMC2387184 18490657

[38] Tully DC Ogilvie CB Batorsky RE Bean DJ Power KA Ghebremichael M. Differences in the selection bottleneck between modes of sexual transmission influence the genetic composition of the HIV-1 founder virus. PloS Pathog (2016) 12(5):e1005619. doi: 10.1371/journal.ppat.1005619 27163788 PMC4862634

[39] Carlson JM Schaefer M Monaco DC Batorsky R Claiborne DT Prince J. HIV Transmission. Selection bias at heterosexual HIV-1 Transm bottleneck Science (2014) 345(6193):1254031. doi: 10.1126/science.1254031 PMC4289910 25013080

[40] Simek MD Rida W Priddy FH Pung P Carrow E Laufer DS. Human immunodeficiency virus type 1 elite neutralizers: individuals with broad and potent neutralizing activity identified by using a high-throughput neutralization assay together with an analytical selection algorithm. J Virol (2009) 83(14):7337–48. doi: 10.1128/JVI.00110-09 PMC2704778 19439467

[41] Walker LM Simek MD Priddy F Gach JS Wagner D Zwick MB. A limited number of antibody specificities mediate broad and potent serum neutralization in selected HIV-1 infected individuals. PloS Pathog (2010) 6(8):e1001028. doi: 10.1371/journal.ppat.1001028 20700449 PMC2916884

[42] McCoy LE Burton DR. Identification and specificity of broadly neutralizing antibodies against HIV. Immunol Rev (2017) 275(1):11–20. doi: 10.1111/imr.12484 28133814 PMC5299474

[43] deCamp A Hraber P Bailer RT Seaman MS Ochsenbauer C Kappes J. Global panel of HIV-1 env reference strains for standardized assessments of vaccine-elicited neutralizing antibodies. J Virol (2014) 88(5):2489–507. doi: 10.1128/JVI.02853-13 PMC3958090 24352443

[44] Ventura JD Beloor J Allen E Zhang T Haugh KA Uchil PD. Longitudinal bioluminescent imaging of HIV-1 infection during antiretroviral therapy and treatment interruption in humanized mice. PloS Pathog (2019) 15(12):e1008161. doi: 10.1371/journal.ppat.1008161 31805155 PMC6917343

[45] Price MA Kilembe W Ruzagira E Karita E Inambao M Sanders EJ. Cohort profile: IAVI's HIV epidemiology and early infection cohort studies in Africa to support vaccine discovery. Int J Epidemiol (2021) 50(1):29–30. doi: 10.1093/ije/dyaa100 32879950 PMC7938500

[46] Umviligihozo G Muok E Nyirimihigo Gisa E Xu R Dilernia D Herard K. Increased frequency of inter-subtype HIV-1 recombinants identified by near full-length virus sequencing in Rwandan acute transmission cohorts. Front Microbiol (2021) 12:734929. doi: 10.3389/fmicb.2021.734929 34690973 PMC8529237

[47] Balinda SN Kapaata A Xu R Salazar MG Mezzell AT Qin Q. Characterization of near full-length Transmitted/Founder HIV-1 subtype d and A/D recombinant genomes in a heterosexual Ugandan population (2006-2011). Viruses (2022) 14(2):334–58. doi: 10.3390/v14020334 PMC8874453 35215928

[48] McGowan E Rosenthal R Fiore-Gartland A Macharia G Balinda S Kapaata A. Utilizing computational machine learning tools to understand immunogenic breadth in the context of a CD8 T-cell mediated HIV response. Front Immunol (2021) 12:609884. doi: 10.3389/fimmu.2021.609884 33679745 PMC7930081

[49] Price MA Rida W Kilembe W Karita E Inambao M Ruzagira E. Control of the HIV-1 load varies by viral subtype in a Large cohort of African adults with incident HIV-1 infection. J Infect Dis (2019) 220(3):432–41. doi: 10.1093/infdis/jiz127 PMC6603968 30938435

[50] Gao F Bonsignori M Liao HX Kumar A Xia SM Lu X. Cooperation of b cell lineages in induction of HIV-1-broadly neutralizing antibodies. Cell (2014) 158(3):481–91. doi: 10.1016/j.cell.2014.06.022 PMC4150607 25065977

[51] Salazar-Gonzalez JF Salazar MG Keele BF Learn GH Giorgi EE Li H. Genetic identity, biological phenotype, and evolutionary pathways of transmitted/founder viruses in acute and early HIV-1 infection. J Exp Med (2009) 206(6):1273–89. doi: 10.1084/jem.20090378 PMC2715054 19487424

[52] Adachi A Gendelman HE Koenig S Folks T Willey R Rabson A. Production of acquired immunodeficiency syndrome-associated retrovirus in human and nonhuman cells transfected with an infectious molecular clone. J Virol (1986) 59(2):284–91. doi: 10.1128/jvi.59.2.284-291.1986 PMC253077 3016298

[53] Baalwa J Wang S Parrish NF Decker JM Keele BF Learn GH. Molecular identification, cloning and characterization of transmitted/founder HIV-1 subtype a, d and A/D infectious molecular clones. Virol (2013) 436(1):33–48. doi: 10.1016/j.virol.2012.10.009 PMC3545109 23123038

[54] Dilernia DA Chien JT Monaco DC Brown MP Ende Z Deymier MJ. Multiplexed highly-accurate DNA sequencing of closely-related HIV-1 variants using continuous long reads from single molecule, real-time sequencing. Nucleic Acids Res (2015) 43(20):e129. doi: 10.1093/nar/gkv630 26101252 PMC4787755

[55] Deymier MJ Ende Z Fenton-May AE Dilernia DA Kilembe W Allen SA. Heterosexual transmission of subtype c HIV-1 selects consensus-like variants without increased replicative capacity or interferon-α resistance. PloS Pathog (2015) 11(9):e1005154. doi: 10.1371/journal.ppat.1005154 26378795 PMC4574710

[56] Deymier MJ Claiborne DT Ende Z Ratner HK Kilembe W Allen S. Particle infectivity of HIV-1 full-length genome infectious molecular clones in a subtype c heterosexual transmission pair following high fidelity amplification and unbiased cloning. Virology (2014) 468-470:454–61. doi: 10.1016/j.virol.2014.08.018 PMC4252503 25243334

[57] Ozaki DA Gao H Todd CA Greene KM Montefiori DC Sarzotti-Kelsoe M. International technology transfer of a GCLP-compliant HIV-1 neutralizing antibody assay for human clinical trials. PloS One (2012) 7(1):e30963. doi: 10.1371/journal.pone.0030963 22303476 PMC3267749

[58] Wei X Decker JM Liu H Zhang Z Arani RB Kilby JM. Emergence of resistant human immunodeficiency virus type 1 in patients receiving fusion inhibitor (T-20) monotherapy. Antimicrob Agents Chemother (2002) 46(6):1896–905. doi: 10.1128/AAC.46.6.1896-1905.2002 PMC127242 12019106

[59] Hare J Macharia G Yue L Streatfield CL Hunter E Purcell A. Direct identification of HLA-presented CD8 T cell epitopes from transmitted founder HIV-1 variants. Proteomics (2021) 21(17-18):e2100142. doi: 10.1002/pmic.202100142 34275180

[60] Hare J Morrison D Nielsen M. Sampling SARS-CoV-2 proteomes for predicted CD8 T-cell epitopes as a tool for understanding immunogenic breadth and rational vaccine design. Front Bioinf (2021) 1. doi: 10.3389/fbinf.2021.622992 PMC9581046 36303758

[61] Keefer MC Gilmour J Hayes P Gill D Kopycinski J Cheeseman H. A phase I double blind, placebo-controlled, randomized study of a multigenic HIV-1 adenovirus subtype 35 vector vaccine in healthy uninfected adults. PloS One (2012) 7(8):e41936. doi: 10.1371/journal.pone.0041936 22870265 PMC3411704

[62] Michelo CM Dalel JA Hayes P Fernandez N Fiore-Gartland A Kilembe W. Comprehensive epitope mapping using polyclonally expanded human CD8 T cells and a two-step ELISpot assay for testing large peptide libraries. J Immunol Methods (2021) 491:112970. doi: 10.1016/j.jim.2021.112970 33529681 PMC8008507

[63] Macharia GN Yue L Staller E Dilernia D Wilkins D Song H. Infection with multiple HIV-1 founder variants is associated with lower viral replicative capacity, faster CD4+ T cell decline and increased immune activation during acute infection. PloS Pathog (2020) 16(9):e1008853. doi: 10.1371/journal.ppat.1008853 32886726 PMC7498102

[64] Morvan MG Teque FC Locher CP Levy JA. The CD8(+) T cell noncytotoxic antiviral responses. Microbiol Mol Biol Rev (2021) 85(2):e00155–20. doi: 10.1128/MMBR.00155-20 33980586 PMC8139528

[65] Makinde J Nduati EW Freni-Sterrantino A Streatfield C Kibirige C Dalel J. A novel sample selection approach to aid the identification of factors that correlate with the control of HIV-1 infection. Front Immunol (2021) 12:634832. doi: 10.3389/fimmu.2021.634832 33777023 PMC7991997

