# Supplementary material for: Assessment of a diverse panel of transmitted/founder HIV-1 infectious molecular clones in a luciferase based CD8 T-cell mediated viral inhibition assay
Source: Front Immunol. 2022 Dec 1;13:1029029. doi: 10.3389/fimmu.2022.1029029 (PMC9751811; doi:10.3389/fimmu.2022.1029029)
Supplement: Supplementary file 1 [file Table_1.docx]

**Supplementary Table 1.** IMC-LucR panel composition

| **Number** | **IMC-LucR Name*** | **IMC-LucR**  **Code ID Name** | **K Number (Unique plasmid identifier)** | **Clade** |
| --- | --- | --- | --- | --- |
| 1 | CH077.t-LucR.6ATRi | N/A | K4472 | B |
| 2 | UG.191955-LucR.6ATRi | UG.191955-LucR.6ATRi | K5531 | A/D |
| 3 | ZM247F_V2-LucR.6ATRi | N/A | K5222 | C |
| 4 | CH106.c-LucR.6ATRi | N/A | K5223 | B |
| 5 | UG.191996-LucR.6ATRi | UG.191996-LucR.6ATRi | K5560 | A/D |
| 6 | Z.235239-LucR.6ATRi | Z4248M_TF-LucR.6ATRi | K4802 | C |
| 7 | UG.275031-LucR.6ATRi | UG.275031-LucR.6ATRi | K5501 | D |
| 8 | Z.305144-LucR.6ATRi | Z.305144-LucR.6ATRi | K5609 | C |
| 9 | NL4-3-LucR.6ATRi | N/A | K5300 | B |
| 10 | UG.193008-LucR.6ATRi | UG.193008-LucR.6ATRi | K5545 | A/D |
| 11 | R.175059-LucR.6ATRi | R977F#49-1-LucR.6ATRi | K5333 | A |
| 12 | UG.191696-14A-LucR.6ATRi | UG.191696-LucR.6ATRi | K5495 | A/D |
| 13 | R.175090-LucR.6ATRi | R3469F -LucR.6ATRi | K5365 | A |
| 14 | R.175019-LucR.6ATRi | R6185M-21-LucR.6ATRi | K5163 | A |
| 15 | KE.210011-LucR.6ATRi | KE.210011-LucR.6ATRi | K5562 | A/D |
| 16 | UG.191947-LucR.6ATRi | UG.191947-LucR.6ATRi | K4815 | A/D |
| 17 | R.175053-LucR.6ATRi | R.175053-LucR.6ATRi | K5445 | A/C |
| 18 | UG.194535-LucR.6ATRi | UG.194535-LucR.6ATRi | K5555 | D |
| 19 | Z.235219-LucR.6ATRi | Z3618M_TF-LucR.6ATRi | K4798 | C |
| 20 | KE.210023-LucR.6ATRi | KE.210023-LucR.6ATRi | K5634 | A/C |
| 21 | UG.191882-LucR.6ATRi | UG.191882-LucR.6ATRi | K4813 | D |
| 22 | Z.235214-LucR.6ATRi | Z331M_TF-LucR.6ATRi | K4796 | C |
| 23 | R.175020-LucR.6ATRi | R57F#03-LucR.6ATRi | K5397 | A |
| 24 | UG.191923-LucR.6ATRi | UG.191923-LucR.6ATRi | K5530 | A/D |
| 25 | CH505.s-LucR.6ATRi | N/A | K4474 | C |
| 26 | R.175014-LucR.6ATRi | R59M#41-LucR.6ATRi | K5396 | A |
| 27 | UG.194289-LucR.6ATRi | UG.194289#15-A7-LucR.6ATRi | K5166 | D |
| 28 | KE.220898-LucR.6ATRi | KE.220898-LucR.6ATRi | K5561 | A/D |
| 29 | Z.305123-LucR.6ATRi | Z.305123-LucR.6ATRi | K5586 | C |
| 30 | UG.193006-LucR.6ATRi | UG.193006-LucR.6ATRi | K5544 | A/C/D |
| 31 | R.175089-LucR.6ATRi | R3584M-LucR.6ATRi | K5364 | A/C |
| 32 | Z.235227-LucR.6ATRi | Z3678M_TF-LucR.6ATRi | K4800 | C |
| 33 | UG.194346-LucR.6ATRi | UG.194346-LucR.6ATRi | K5547 | A/D |
| 34 | Z.235092-LucR.6ATRi | Z1123M-LucR.6ATRi | K5336 | C |
| 35 | UKZN498-LucR.6ATRi | UKZN498-LucR.6ATRi | K5611 | C |

*Referred name in the text
